# Delivering Therapeutics to Glioblastoma: Overcoming Biological Constraints

**DOI:** 10.3390/ijms23031711

**Published:** 2022-02-02

**Authors:** Elza N. Mathew, Bethany C. Berry, Hong Wei Yang, Rona S. Carroll, Mark D. Johnson

**Affiliations:** Department of Neurological Surgery, University of Massachusetts Medical School, 55Lake Avenue North, Worcester, MA 01655, USA; elzaneelima.mathew@gmail.com (E.N.M.); bethany.berry@umassmed.edu (B.C.B.); hongwei.yang@umassmed.edu (H.W.Y.); rona.carroll@umassmed.edu (R.S.C.)

**Keywords:** glioblastoma, brain tumor, drug delivery

## Abstract

Glioblastoma multiforme is the most lethal intrinsic brain tumor. Even with the existing treatment regimen of surgery, radiation, and chemotherapy, the median survival time is only 15–23 months. The invasive nature of this tumor makes its complete removal very difficult, leading to a high recurrence rate of over 90%. Drug delivery to glioblastoma is challenging because of the molecular and cellular heterogeneity of the tumor, its infiltrative nature, and the blood–brain barrier. Understanding the critical characteristics that restrict drug delivery to the tumor is necessary to develop platforms for the enhanced delivery of effective treatments. In this review, we address the impact of tumor invasion, the molecular and cellular heterogeneity of the tumor, and the blood–brain barrier on the delivery and distribution of drugs using potential therapeutic delivery options such as convection-enhanced delivery, controlled release systems, nanomaterial systems, peptide-based systems, and focused ultrasound.

## 1. Introduction

Glioblastoma multiforme (GBM) is the most common primary brain malignancy in adults, accounting for more than 50% of intrinsic brain tumors [1]. According to the World Health Organization (WHO) classification, GBM is a grade IV glioma, highly invasive with a five-year survival rate of less than 5% [2,3]. This highly aggressive disease presents a very poor prognosis. The median survival time from diagnosis of the tumor is approximately 15–23 months. The incidence of GBM is 3.19 per 100,000 persons in the United States with a median age of 64 years [4,5,6,7,8].

Widely recognized risk factors associated with GBM occurrence are exposure to high-dose ionizing radiation and certain genetic syndromes, including neurofibromatosis type 1 and Li-Fraumeni syndrome [2,9,10]. The occurrence of GBM is higher in males and among individuals 50 years of age and older [5].

GBM is characterized by rapid cell proliferation and extensive invasion of tumor cells into the surrounding brain, making complete removal of the tumor impossible [11]. These features lead to a high recurrence rate, even with the current treatment regimen of maximum safe surgical removal, radiation therapy and temozolomide chemotherapy. Intratumoral molecular heterogeneity, presence of the blood–brain barrier, and tumor immune evasion via local immunosuppression limits the success of existing therapies [12]. Numerous drug therapies directed against GBM have shown promising results in in vitro assays, but all have had limited success in vivo. This is due in part to the diffuse, heterogeneous nature of the tumor, and the blood–brain barrier that limits the ability of many drugs to enter the brain parenchyma. Understanding the critical factors that restrict drug delivery to the brain is necessary to develop platforms for the enhanced delivery of effective drugs.

**Figure 1:**
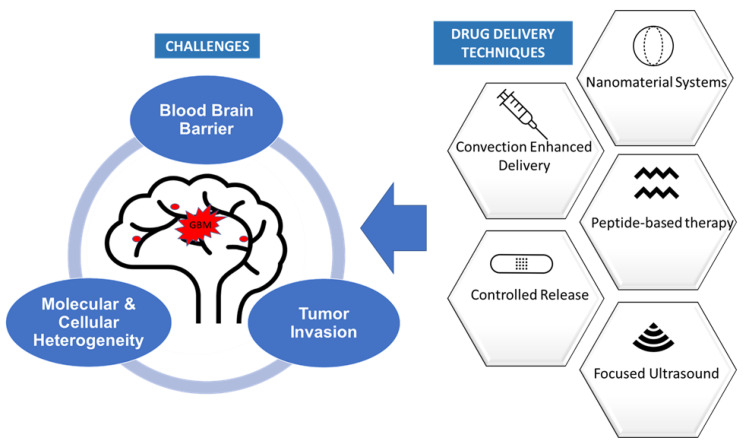
Challenges to delivery of therapeutics to glioblastoma and the currently available drug delivery techniques.

In this review, we discuss the impact that molecular and cellular tumor heterogeneity, tumor dispersion and the blood–brain barrier have on the delivery and distribution of pharmacological agents to GBM. The currently available chemotherapeutic delivery systems designed to overcome these constraints are also discussed (Figure 1). We will focus on the use of convection-enhanced delivery, controlled release systems, nanomaterial systems, peptide-based therapeutics and focused ultrasound (FUS) for the treatment of this aggressive tumor.

### 1.1. Molecular Heterogeneity

GBM is characterized by molecular heterogeneity within a single tumor in addition to inter-patient heterogeneity. Molecular heterogeneity underlies the cellular heterogeneity in GBM, i.e., the differences between cell types within a tumor [13]. This molecular heterogeneity may be responsible for differences in individual patient responses to therapy and prognosis as well as the failure of targeted therapies [14,15]. Common gene mutations include IDH1/2 mutations, O6-methylguanine DNA methyltransferase (MGMT) promoter methylation, co-deletion of 1p and 19q, and EGFR amplification/truncation. Primary GBM frequently exhibits epidermal growth factor receptor (EGFR) over expression, PTEN (MMC-I) mutation or deletion, CDKN2A (p16) deletion, and MDM2 amplification. Secondary GBM predominantly has IDH1, ATRX and TP53 mutations [16]. Based on differences in genetic alterations and the expression of EGFR, NF1, PDGFRA/IDH1, PI3K and other key genes, primary GBM can be classified into four subtypes: proneural, neural, classical, and mesenchymal, with each subtype varying in its gene expression signature [17]. Secondary GBMs predominantly have IDH1 (or less commonly IDH2) mutations that have a proneural gene expression signature and a better prognosis than GBMs with wild-type IDH1/IDH2.

In addition to the molecular heterogeneity described above, GBM cells are morphologically and functionally heterogeneous. Single-cell RNA-Seq studies of individual tumor cells have revealed that GBM tumor cells exist in multiple stages of differentiation [18]. Other studies have shown that astrocyte-like GBM cells can transdifferentiate to become endothelial-like cells [19,20]. As a result of this extraordinary cellular heterogeneity, individual GBM cells may be more or less replicative, invasive, or sensitive to radiation or chemotherapy [21].

### 1.2. Blood–Brain Barrier

The blood–brain barrier is composed of a highly specialized circuit of blood vessels that are lined by brain microvascular endothelial cells (BMEC), the cell–cell junctions between which restrict the entry of potentially harmful substances (including systemically administered therapeutics) into the brain. The BMECs are surrounded by pericytes, astrocytes and the basal membrane [22,23]. This is indeed a protective layer safeguarding the brain from damaging agents in the systemic circulation and keeping CNS homeostasis to allow proper neuronal function [24]. The two major features of the blood–brain barrier are: (1) the presence of tight junctions limiting paracellular transport, and (2) reduced fenestrations and transport vesicles limiting transcellular transport. Small molecules need to be less than 400 Da in size and lipid soluble in order to cross the barrier [25].

Disruption of the blood–brain barrier (BBB) is observed in glioma tumor regions. Unfortunately, this degree of BBB disruption is not sufficient to allow for the ability of therapeutics to reach this diffusely infiltrating tumor [26,27]. Biochemical and physical methods can be used to further increase the permeability of this biological barrier. Ion channel activators such as ATP-sensitive and calcium-activated potassium channel activators, phosphodiesterase 5 (PDE5) inhibitors, bradykinin type 2 receptor activators, adenosine 2A receptor (A2AR) agonists, papaverine, and certain microRNAs represent biochemical approaches to biochemical modulation of the BBB. PDE5 inhibitors decrease cGMP degradation and increase vesicular transport, thereby increasing BBB permeability within the tumor region. Potassium channel activators downregulate tight junction protein expression and increase the formation of pinocytotic vesicles. Bradykinin activators increase transcytosis and modulate tight junction protein expression and cGMP synthesis. A2AR agonists and papaverine downregulate tight junction protein expression. MicroRNAs such as miR-132-3p increase blood–brain barrier permeability by increasing transcytosis. Mannitol, an osmotic agent, is widely used to disrupt the blood–brain barrier by vasodilatation and shrinkage of endothelial cells [28,29]. Physical strategies for modulating BBB permeability include the application of electromagnetic pulses, laser-induced thermal therapy, radiotherapy, and focused ultrasound (FUS). All these methods downregulate the expression of tight junction proteins. FUS is also involved in increasing transcytosis [22,30].

## 2. Methods of Drug Delivery to Glioblastoma

In this review, we will discuss the application of controlled release systems, convection-enhanced delivery, nanomaterial systems, peptide-based therapeutics, and focused ultrasound for drug delivery to glioblastoma, overcoming the obstacles posed by GBM molecular and cellular heterogeneity, GBM cell invasion/dispersal and the blood–brain barrier.

### 2.1. Controlled-Release Systems

Implantable drug release systems enable the direct delivery of therapeutic agents to the tumor site, circumventing the need to cross the blood–brain barrier. Drug-loaded biocompatible materials such as drug-impregnated gels can be designed so that they release low doses of drugs at the tumor site over a prolonged period of time.

Implantable controlled-release delivery systems can be constructed with degradable or non-degradable polymers. Between these two options, biodegradable polymers are more commonly used [31]. One such biodegradable polymer delivery system that has been used clinically for GBM is the Gliadel^®^ wafer. This is a biodegradable polymer wafer loaded with the chemotherapeutic drug BCNU. It is the only FDA-approved drug delivery implant for treating GBM [11]. Gliadel was approved by the FDA in 1996 for recurrent GBM and later in 2003 for upfront treatment of malignant glioma [31,32,33]. In another study, biodegradable wafers were created for the combined delivery of temozolomide and carmustine in a rat glioma model. This approach increased the median survival of the animals significantly, with 25% of the animals living long term > 120 days [34]. Biodegradable polymer implants releasing rapamycin were found to increase survival both in the presence and absence of radiation therapy in a rat malignant glioma model [35]. The rigid structure of these systems has limited drug loading capacity and they can be dislodged from the original site of implantation. In addition, the wafer systems also led to the occurrence of seizures, intracranial hypertension, meningitis, cerebral edema, and impaired wound healing in neurosurgical patients [11].

A pH-responsive carboxymethylcellulose biopolymer system was used to deliver the chemotherapeutic drug, rhodamine B encapsulated in multiple emulsions to glioblastoma cells in vitro [36]. In a mouse GBM tumor resection model, hydrogel-based co-delivery of the chemotherapeutic agents paclitaxel and temozolomide enhanced survival [37]. Hydrogels can adapt their shape while nevertheless retaining sufficient drug capacity [11]. The use of multiple emulsions and hydrogels avoided the limitations seen with the rigid structure of wafer implants. However, the intrinsic hydrophilic nature of hydrogels does not allow for the effective delivery of hydrophobic therapeutic agents [38].

With sustainable release of therapeutic agents over time, implantable controlled-release systems limit the local GBM recurrence after resection by interacting with the tumor cells at the resection site. However, these systems are not only limited by a variety of side effects as listed above, but also by poor drug distribution to distant tumor cells that have migrated in the normal brain parenchyma. Consequently, these implantable local delivery systems have limited ability to affect infiltrating GBM cells distant to the site of implantation.

### 2.2. Convection-Enhanced Delivery

Convection-enhanced delivery (CED) is a catheter-based drug delivery method that depends on pressure gradients rather than diffusion to deliver therapeutic agents into brain tumors. This technique involves the stereotactic insertion of one or more catheters into the tumor. The catheters are connected to a syringe/drug delivery pump that maintains a positive pressure gradient that promotes the distribution of higher volumes of drug over a larger area. Ensuring direct intratumoral drug delivery independent of drug concentration or diffusivity of the infusate, CED allows the use of large volumes of the drug at lower and less-toxic doses [39]. The ratio between the volume of distribution and the volume of infusion is a key factor determining the optimum flow rate and success of this technique.

CED of temozolomide, the standard cytostatic drug for the treatment of GBM, combined with subcutaneous immunizations with irradiated cancer cells had a synergistic effect on tumor growth and overall survival in a mouse-GL261 glioma model. However, the same protocol did not display synergy in the KR158 mouse glioma model, which is resistant to radiation and chemotherapy. In the latter model, CED of temozolomide increased survival [40]. By initiating antitumor immune response, irradiated cells may help to overcome intratumoral immunosuppression and enhance the antitumor activity of temozolomide.

In a phase I clinical trial, CED was used to infuse carboplatin to patients with WHO grade III astrocytomas or grade IV gliomas (GBM). This approach increased median overall survival without any systemic toxicity [41]. This was the first clinical trial to demonstrate that CED of carboplatin into the brain is safe. Oral or intravenous administration of platinated drugs does not produce effective concentrations in the brain and has been associated with systemic toxicity [42].

MRI-guided CED of iron oxide nanoparticles (IONP) conjugated to epidermal growth factor receptor deletion mutant III antibody (EGFRVIIIAb) showed significant increase in survival in a mouse GBM model [43]. Binding of EGFRvIIIAb-IONP conjugate to target cells was evaluated by changes in the MRI signal. IONP is a theranostic nanoparticle which has imaging properties as well as anticancer activity. EGFRvIIIAb is a tumor-specific cell surface protein. The experimental animal groups treated with EGFRvIIIAb-IONP conjugate as well as EGFRvIIIAb alone had a single survivor after 120 days. This study demonstrated the feasibility of conjugating biological agents or ligands that bind to tumor-specific proteins along with concomitant imaging to ensure targeted distribution of the therapeutic agent.

Direct delivery of anticancer agents in large volumes and at less toxic doses makes CED an attractive drug delivery method for GBM. Implantable catheters can be used to repeat CED at required intervals, but the efficacy of this approach may be limited by the highly invasive nature of the tumor with GBM cells dispersed to distant sites [44]. Challenges in maintaining the ratio between the volumes of distribution and infusion, flow rate, formation of air bubbles, infusate reflux along the catheter, and the variability in the tumor tissue composition can limit the establishment of the required pressure gradient, thereby limiting the efficacy of CED [45].

### 2.3. Nanomaterial Systems

Nanomedicine is an emerging candidate for cancer therapy that uses a variety of different types of nanocarriers such as lipid-based, polymer-based, inorganic, viral, and drug-conjugated nanoparticles [46]. Small size, high surface-to-volume ratio and other physico-chemical parameters make nanoparticles unique [47]. Accentuated drug delivery along with potential applications in diagnosis and imaging makes nanomedicine an appealing approach to cancer therapeutics [48].

Nanomaterial systems for drug delivery improve GBM tumor targeting mainly by promoting drug diffusion through the blood–brain barrier and by the enhanced permeability and retention (EPR) effect. The EPR effect is dependent on the highly angiogenic nature of GBM where leaky vasculature is commonly present [49]. Because of this phenomenon, nanoparticles can passively modulate the biodistribution of loaded molecules and increase their accumulation in cancer tissues with leaky vasculature [50,51,52]. The efficacy of antitumor drug delivery via nanoparticles is also enhanced by several additional mechanisms, including increased cellular internalization, activation of immune cells, and reactive oxygen species (ROS) production [53]. Surface functionalization of a nanoparticle with an active principle, a targeting agent, and a compound for detection will increase the functional range of the nanomaterial drug delivery targeting biomolecules, thereby enabling both drug delivery and disease diagnosis [54]. The long half-life and improved cellular uptake and biodistribution increase the appeal of nanomaterial systems for tumor therapy [49].

The delivery of tumor antigens and adjuvants using nanoparticles as a vehicle has been shown to increase the efficacy of immune therapy against GBM [55,56,57]. Different types of nanomaterials, including polymeric nanoparticles or lipid-based nanoparticles, may also be used to deliver nucleic acids intended for tumor therapy [49]. A poly(ε-caprolactone) (PCL)-based nanoparticle system was used to deliver the natural growth modulating tripeptide GHK (glycyl-L-histidyl-L-lysine) to human GBM cells in vitro, thereby reducing GBM cell viability to nearly 65%. However, this approach did not show anticancer activity at concentrations less than 20 mg/mL [58]. GHK is a natural tripeptide with a varied array of biological activities including anticancer, antioxidant, anti-inflammatory, skin remodeling, wound healing, etc. [59]. The anti-GBM activity of GHK demonstrated in this study in vitro requires further validation in vivo.

A nanobubble-based theranostic system consisting of intravenously administered iron-platinum nanoparticles loaded with doxorubicin and surface-functionalized with transferrin (to allow for tumor targeting) reduced glioma growth in a mouse model by almost 70%. The nanobubbles were burst by exposing to high intensity focused ultrasound (HIFU) to bypass the blood–brain barrier by generating a cavitation effect. This single nanocomposite, combining nanomaterials, chemotherapeutic agents and MRI contrast agents all together for the first time, was able to cross the blood–brain barrier, target the tumor cells and allow imaging of the brain tumor. This multimodal system is an example of combining several strategies to enhance the success rate of therapy [60].

The physicochemical properties of nanomaterials enable them to deliver therapeutic agents to the brain via drug encapsulation or by surface modification. The clinical use of the nanomaterial drug delivery systems is limited by the poorly controlled accumulation and distribution of particles in and around the specific target site [61,62]. The use of multiple therapeutic strategies in combination with nanocarriers may improve the success rate of targeted tumor therapies. However, the potential toxic effects of nanoparticle systems due to aggregation upon introduction into biological systems remains poorly understood. The effect of protein corona formation around nanoparticles and its relevance regarding their biological activity is also being extensively investigated [63].

### 2.4. Peptide Based Therapeutics

There are three main types of peptide-based therapeutics—tumor homing peptides, peptides targeting aberrant cellular signaling pathways and cell-penetrating peptides [64]. GBM cells have increased expression of membrane proteins that are responsible for cellular function and maintenance, protein synthesis, intercellular signaling, cell movement, and antigen presentation [8,65]. Tumor homing peptides can bind to specific molecular targets on the surface of GBM cells and will be taken up by the cells by endocytosis [66]. The binding process of these peptides is faster than antibodies. These can also be used for in vivo tumor imaging. Binding of some of these peptides can also enhance or antagonize signal transduction pathways in cancer cells or tumor tissues. Peptides and their derivatives targeting aberrant cellular signaling pathways can improve the efficacy of tumor therapy by increased selectivity in their interaction with the oncogenic pathways [64]. Cell-penetrating peptides (CPPs) are small, basic, positively charged peptide derivatives that can pass through the cell membrane [64,67].

Highly selective tumor-targeting peptides obtained using a biopanning phage display library directed against GBM cells were able to cross the BBB and deliver the oncolytic virus VSVΔM51 to GBM in a mouse model in vivo [68]. These peptides, when delivered in combination with gadolinium, also enabled the visualization of the tumors via MRI. The use of peptides directed against multiple targets provides a mechanism for addressing the heterogeneity of the tumor while nevertheless allowing for tumor specific oncolytic virus delivery.

Self-assembled spherical nanoparticles containing a peptide probe (Cy5.5-SAPD-99mTc) that combines tumor homing ability with mitochondria targeting was found to have promising theranostic possibilities owing to the enhanced apoptosis in GBM cells coupled with imaging functionality [69]. Incorporating both tumor-homing and mitochondria-targeting components helps to increase the specificity of drug delivery.

Peptide derivatives of rabies virus glycoproteins, RVG29 and RVG15-liposome, were used to deliver anticancer chemotherapeutic docetaxel nanoparticles and paclitaxel-cholesterol to glioma-bearing ICR mice with a positive effect on animal survival [70,71]. The RVG peptides target the nicotinic acetylcholine receptor, the increased expression of which is noted in the hypoxic and ischemic conditions within the tumor microenvironment. Administration of RVG peptides thus aids in tumor-specific chemotherapeutic delivery.

WSW (also called PhrCACET1) is a tumor-targeting peptide (derived from Clostridium acetobutylicum) that was fused to paclitaxel nanosuspensions and used to target GBM cell membranes in a glioma mouse model. The use of WSW induced apoptosis and prolonged survival of the animals [72]. By combining BBB penetration with tumor targeting, this biomimetic drug delivery system has enhanced tumor-targeting specificity.

The use of polydopamine (PDA)-coated zein-curcumin nanoparticles functionalized with the peptide G23 inhibited cell proliferation and migration in glioma cells in vitro [73]. Here, the peptide G23 binds to ganglioside GM-1 and enables crossing of the BBB. The anti-inflammatory, antimicrobial, and anticancer activities of curcumin have been widely demonstrated [74,75,76].

A dual peptide nanocomplex created by combining SynB3 (a cell penetration peptide) with PVGLIG (an MMP-2 sensitive peptide) and paclitaxel inhibited cell migration and invasion in multiple GBM cell lines, suppressed GBM tumor growth in vivo, and increased overall survival in a mouse model of GBM [77]. The aberrant expression of matrix metalloproteinases (MMPs) has been widely reported in tumors, and the addition of an MMP-sensitive peptide increased the tumor specificity of the drug cargo in this system.

To enhance the membrane permeability of peptides, membrane receptors such as low-density lipoprotein receptor, IL-4 receptor, and transferrin receptor which are abundantly expressed on GBM cells have been used to direct the delivery of tumor-homing peptides to brain malignancies, utilizing receptor-mediated transcytosis [78,79,80]. Peptide-mediated drug delivery is limited by the poor in vivo stability due to the proteolytic degradation of peptides in the circulation when administered systemically. In addition, the short half-life of peptides results in limited bioavailability. This can be overcome by chemical modification or conjugation with macromolecules or nanocarriers with greater target specificity [64]. Identifying additional GBM-targeting peptides is needed to further exploit the benefits of this mode of drug delivery.

### 2.5. Focused Ultrasound

Focused ultrasound (FUS) is an image-guided, noninvasive method to transiently open the blood barrier, thereby enhancing the efficacy of therapeutic delivery to GBM. FUS can be used to reversibly disrupt the BBB without irreversible tissue damage [81]. The use of FUS in combination with circulating microbubbles works by creating mechanical cavitation effects that transiently disrupt the BBB [82]. In addition to the cavitation effects and thermal ablation, FUS may also work via immunomodulation [83]. Future concurrent application of noninvasive FUS along with other modes of therapeutic drug delivery to GBM is promising.

By increasing BBB permeability, FUS can enhance the delivery of varied therapeutic agents to the tumor. For example, FUS-induced disruption of the BBB enhanced the local delivery of temozolomide to tumors and increased the overall survival of rats harboring experimentally induced gliomas [84]. FUS application followed by BCNU administration resulted in a reduced rate of tumor growth and improved survival in rats [85]. In a mouse model bearing temozolomide-resistant glioma, low-intensity fluorescent ultrasound (LIFU) was used to deliver a liposomal O6-(4-bromothenyl)guanine (O6BTG) derivative that inactivates MGMT [86]. Because MGMT enhances DNA repair in tumor cells, MGMT silencing has been associated with more favorable outcomes after temozolomide treatment [87].

The use of imaging techniques along with FUS increases the rate of drug delivery to specifically identified tumor tissues. MRI guided FUS (MRgFUS) was used to achieve a higher tissue delivery of liposome-encapsulated doxorubicin in rats, temozolomide in mice, and cisplatin-conjugated gold nanoparticles in mice [84,88,89]. In an additional study, MRgFUS was used to deliver the intravenously administered monoclonal antibody, trastzumab, to Her2-positive intracranial metastases in breast cancer patients without any adverse events [90].

Using this noninvasive technique, relatively low systemic doses of therapeutic agents can be used, thereby reducing systemic toxic effects [91]. Transient application of FUS only results in a transient opening of the BBB and does not lead to long-term BBB defects [25]. FUS has the potential to enhance therapeutic drug efficacy against GBM because it is noninvasive and provides reproducible enhancements in drug delivery in early investigational studies. Clinical trials using FUS in GBM are currently underway [81,92,93]. Nevertheless, FUS is not immune to side effects which include edema, intracerebral hemorrhage, and uncontrolled thermal injury in brain [94].

## 3. Conclusions

Molecular and cellular heterogeneity, GBM cell dispersal and the BBB are critical constraints limiting the efficacy of anti-GBM drug therapy. Applications of CED, controlled-release systems, nanomaterial systems, peptide-based therapeutics and focused ultrasound for drug delivery to tumor enhance survival with reduced toxicity in animal studies (Table 1). Despite currently available treatments, the highly invasive GBM continues to be a deadly disease without cure in patients. Therefore, clinical trials that combine currently available therapies with the novel drug delivery approaches discussed here may enhance the effectiveness of molecular therapeutics in GBM.

## Figures and Tables

**Table 1:** Summary of the methods of drug delivery to GBM with the examples discussed in the text.

Method of Drug Delivery	Specific Examples
Controlled release systems	Gliadel [11,31,32,33]
	Biodegradable wafers for the combined delivery of temozolomide and carmustine [34]
	Biodegradable polymer implants releasing rapamycin [35]
	Carboxymethylcellulose biopolymer system delivering rhodamine B [36]
	Hydrogel based co-delivery of paclitaxel and temozolomide [37]
Convection enhanced delivery	Temozolomide [40]
	Carboplatin [41]
	Iron oxide nanoparticles conjugated to epidermal growth factor receptor deletion mutant III antibody (EG-FRVIIIAb)/MRI-guided [43]
Nanomaterial Systems	Poly(ε-caprolactone) (PCL) based nanoparticle system to deliver the natural growth modulating tripeptide GHK (glycyl-L-histidyl-L-lysine) [58]
	Nanobubble-based theranostic system consisting of intravenously administered iron-platinum nanoparticles loaded with doxorubicin and surface-functionalized with transferrin [60]
Peptide based therapeutics	Tumor targeting peptides delivering deliver the oncolytic virus VSVΔM51, in combination with gadolinium [68]
	Self-assembled spherical nanoparticles containing a peptide probe (Cy5.5-SAPD-99mTc) with mitochondria targeting [69]
	Peptide derivatives of rabies virus glycoproteins, RVG29 and RVG15-liposome, delivering anticancer chemotherapeutic docetaxel nanoparticles and paclitaxel-cholesterol [70,71]
	WSW (also called PhrCACET1) peptide fused to paclitaxel nanosuspensions [72]
	Use of polydopamine (PDA)-coated zein-curcumin nanoparticles functionalized with the peptide G23 [73]
	Dual peptide nanocomplex created by combining SynB3 (a cell penetration peptide) with PVGLIG (an MMP-2 sensitive peptide) and paclitaxel [77]
Focused ultrasound	Temozolomide [84]
	BCNU [85]
	Liposomal O6-(4-bromothenyl)guanine (O6BTG) [86]
	Liposome-encapsulated doxorubicin [88]
	Cisplatin conjugated gold nanoparticles [89]
	Trastzumab [90]

## Data Availability

Not applicable.

## Abbreviation

GBM	Glioblastoma multiforme
WHO	World Health Organization
IDH	Isocitrate dehydrogenase
EGFR	Epidermal growth factor receptor
MGMT	O6-methylguanine DNA methyltransferase
PTN	Pleiotrophin
CDKN2A	Cyclin Dependent Kinase Inhibitor 2A
MDM2	Mouse double minute 2 homolog
NF1	Neurofibromatosis type 1
PDGFRA	Platelet Derived Growth Factor Receptor Alpha
GSC	Glioblastoma stem cells
NSC	Neural stem cell
BEMC	Brain microvascular endothelial cells
ATP	Adenosine triphosphate
PDE5	Phosphodiesterase 5
FUS	Focused ultrasound
CED	Convection enhanced delivery
EPR	Enhanced permeability and retention
ROS	Reactive oxygen species
PCL	Poly(ε-caprolactone)
GHK	glycyl-L-histidyl-L-lysine
CPP	Cell penetrating peptide
PDA	Polydopamine
MMP	Matrix Metalloproteinase
MRI	Magnetic resonance imaging
MRgFUS	Magnetic resonance guided focused ultrasound
LIFU	Low intensity fluorescent ultrasound
HIFU	High intensity fluorescent ultrasound
